# Validating obstetric triage systems, what are we really measuring - A modified Delphi process introducing outcome measures for obstetric emergency triage systems

**DOI:** 10.1186/s12884-025-07476-5

**Published:** 2025-04-02

**Authors:** Linnéa Lindroos, Erica Ernstad, Verena Sengpiel

**Affiliations:** 1 Department of Obstetrics and Gynaecology, Region Västra Götaland, Sahlgrenska University Hospital, Diagnosvägen 14, 416 85 Gothenburg, Sweden; 2 Department of Obstetrics and Gynaecology, Institute of Clinical Sciences, Sahlgrenska Academy, University of Gothenburg, Gothenburg, Sweden

**Keywords:** Triage, Emergency care, Validity, Delphi method, Obstetric, Acuity

## Abstract

**Background:**

Obstetric emergency triage is more complex than general emergency triage, since the pregnant woman, the fetus and labour status all must be assessed. It is a relatively new branch of triage and is not an integrated part of obstetric emergency care in Sweden. As in general emergency triage, there is no definition of true acuity for obstetric emergency patients. This makes validation of triage systems difficult and results in unclear capacity to identify patients requiring urgent attention. Predominately applied surrogate outcome measures do not reflect acuity at the time of triage and are often affected by organisational factors. The study aims to develop a set of weighted surrogate outcome measures representing acuity at the time of triage, enabling construct validation of obstetric triage systems.

**Methods:**

A four-round modified Delphi process was performed at a single tertiary obstetrics department. Seven obstetricians and three midwives participated in round 1, while only obstetricians participated in rounds 2-4 based on the profession’s competence. The consensus level for rounds 2-4 was predefined at 100%.

**Results:**

A set of 31 immediate obstetrician-initiated interventions at the emergency department, for a patient presenting with an urgent condition, were defined. The interventions reflect acuity level at the time of triage and with minimum interference or influence by context. The outcomes were weighted at three levels, stratifying urgency in the most severe presentations of these conditions.

**Conclusion:**

As true acuity in a patient seeking emergency care has not been defined, outcome measures reflecting true acuity at the time of triage should be applied when validating triage systems. Previous studies on validity in obstetric triage systems are scarce and inconclusive regarding internal and external validity. The outcome measures developed in this study may serve as a template for validating obstetric triage systems implemented in similar contexts.

**Supplementary Information:**

The online version contains supplementary material available at 10.1186/s12884-025-07476-5.

## Introduction

Triage aims at identifying and prioritising patients with urgent or acute conditions, enabling timely interventions in order to improve clinical outcome or prevent deterioration in patients seeking emergency care [1]. The concept of emergency triage is relatively new in obstetric emergency care. Currently, there are approximately ten different obstetric triage systems (OTSs) worldwide [2–7]. OTSs differ from other triage systems as they are adapted to physiological changes during pregnancy as well as pregnancy-related and pregnancy-specific complaints. Moreover, OTSs not only evaluate the pregnant woman, but also the fetus and labour status. These characteristics, such as applying the use of adapted vital sign parameters, are a prerequisite for correct and safe prioritisation of obstetric emergency patients [8–11]. Applying non-OTSs in the initial assessment of obstetric patients seeking emergency have been shown to threaten patient safety [11].

Although triage is imperative in emergency care, a standard for validating triage systems remains to be established [12]. This impedes assessment of both external and internal validity. The systems’ true ability to identify patients with urgent medical conditions is consequently unknown.

### Triage process

The triage process is supported by structured triage systems and is successful if the required intervention is provided in a timely manner. Despite the extensive use of triage systems in both non-obstetric and obstetric clinical practice, there are surprisingly few studies evaluating their performance, thereby resulting in limited scientific evidence and an unclear effect on patient outcome [12, 13]. Nevertheless, triage is deemed crucial for prioritisation according to medical urgency level and thus for ensuring patient safety [14–16]. It is also essential to manage the everlasting challenge of overcrowding in emergency departments (EDs), facilitating allocation of limited resources [14, 17, 18].

### Validating triage systems

Validity is defined as a system’s ability to truly measure what it is supposed to measure.

*Criterion validity* reflects the true validity and is assessed by a system’s correlation to a “gold standard”, for example by comparing a new system to a pre-existing validated system. There is, at date, no established golden standard for measuring acuity in obstetric emergency patients.

*Construct validity* uses surrogate outcome measures, identified by expert consensus or chosen by convenience, in the absence of a “gold standard” [19]. *Face validity* is an informal and subjective form of validity, based on the expertise of the experts developing a new system [20].

A generally accepted definition of validity within the triage field is *“the degree to which the measured acuity level reflects the patient’s true acuity at the time of triage”* [18]. As there is no definition of true acuity, construct validity with different surrogate outcome measures is assessed. Mortality in the ED, admission to hospital or intensive care unit (ICU), length of stay (LOS) in the ED, resource utilisation and costs might serve as surrogate outcomes [13].

The appropriateness of such outcome measures and the extent to which they reflect acuity at the time of triage have repeatedly been debated [13, 18–21]. The outcome measures are highly affected by local factors such as ED organisation, available resources and the situation in related in-house departments [22]. The rationale for applying the outcome measures and a description of their context is rarely presented, and reference standards or measures have yet to be established [19, 23]. Furthermore, the purpose of the triage process is to establish acuity at the time of triage, not to predict clinical outcome.

Without defined outcome measures, validating triage systems and their ability to accurately triage patients seeking emergency care may jeopardize patient safety, putting patients at risk of being falsely assessed and hence, receiving inadequate and/or untimely care [9, 11, 24]. Indeed, two reviews concerning OTS validity have revealed extensive research gaps. Apart from the Maternal Fetal Triage Index, for which content validity was tested during development, the authors conclude that no adequate testing for any form of validity has been undertaken for existing OTSs [7, 12]. Later, several types of validity testing for the Iranian OB Triage Index were presented. However, the methodology may be questioned, as the above-mentioned, previously debated outcome measures were used for evaluation; furthermore, criterion validity was assessed by comparison with a triage system previously deemed inappropriate for the context [4].

### The Delphi method

Today, there are no established acuity measures for validating OTSs. In 2009 Henriëtte A. Moll proposed a process for validating triage systems, starting with “*deciding on the best proxy for prognosis, ‘‘the reference standard’’ for the urgency classification”* [20] and it has repeatedly been suggested that the consensus-focused, multistage Delphi method would be ideal for establishing a reference standard [18, 20, 25, 26]. The Delphi method is defined as “*a structured process that uses a series of questionnaires or rounds to gather information. Rounds are held until group consensus is reached”* [26]. The method structures knowledge attained by extensive experience rather than research-based information, when such is lacking. A modified Delphi method includes a panel of experts, combining questionnaires and physical meetings to rate indicators or items. Items selected via a Delphi method have high face validity, a prerequisite for further validity testing [26].

The aim of this study was to establish surrogate outcome measures for acuity at the time of triage based on an expert modified Delphi process. These outcomes may be used in future studies to validate obstetric triage systems in contexts similar to the Swedish context. The method described may save as a template for developing surrogate outcome measures in other contexts.

## Method

The study comprises a modified Delphi method process at a single department of obstetrics in Sweden. The aim was to develop a set of weighted surrogate outcome measures representing acuity at the time of triage for construct validation of OTSs. Potential outcome measures should evaluate the degree of acuity at the time of triage reflected by the ED physician’s assessment of the patient and immediate interventions, and with minimum interference or influence by context.

### Setting

The Department of Obstetrics at Sahlgrenska University Hospital/Östra is a tertiary care unit, including an obstetric ED with implemented obstetric emergency triage. The department annually manages approximately 10,000 births and 14,000 emergency visits, from gestational week 18 and up to 12 weeks postpartum. It serves as a county hospital for slightly over 0.5 million inhabitants and is the referral hospital for three county hospitals serving an additional 1.2 million inhabitants. The closest department of obstetrics is 70 km away. The obstetric ED applies the Gothenburg Obstetric Triage System (GOTS), a five-level OTS using symptoms and vital sign parameters for patient assessment. Initial management recommendations include for example electrocardiograms (ECG), cardiotocogram (CTG) and laboratory tests [27]. Midwives perform triage, supported by GOTS, and thereafter assess and manage patient complaints within their profession’s competence e.g. rupture of membranes, start of delivery and first assessment of reduced fetal movements. Midwives manage approximately 40 % of patients seeking emergency care whereas the rest are assessed and managed by an obstetrician [27]. The department manages low- and high-risk pregnancies. In addition to the midwife and auxiliary nurse staff that are the primary caregivers for uncomplicated deliveries, there are always two senior obstetricians and two trainee obstetricians on site during on-call hours. There is 24/7 operating-theatre accessibility and neonatal care for births from gestational week 22+0. For patient demographics, please see Table 1.

**Table 1:** Patient demographics for Sahlgrenska University Hospital, 2022

	**(n)**	**(%)**
Deliveries	8902	100
Primipara	4240	47.6
Smokers at first contact with primary maternity care		
Yes	192	2.2
Missing	196	2.2
BMI		
• ≤ 18.5	152	1.8
• 18.5 – 24.9	4629	55.0
• 25 – 34.9	3267	38.8
• 35.0 – 39.9	267	3.2
• ≥ 40	106	1.3
Missing	481	5.4
Number of prenatal visits		
• ≤ 7	1407	15.9
• 8 – 12	6169	69.5
• ≥ 13	1300	14.6
Missing	26	0.3
Region of origin		
• Sweden	5483	69.1
• Other Nordic countries	62	0.8
• Other European countries	667	8.4
• Other countries	1727	21.8
Missing	963	10.8

Average age for first delivery was 31.1 years.

### Participants

Eligible participants (seven obstetricians and seven midwives) were purposefully invited, based on their extensive experience in both obstetrics and obstetric emergency triage. The midwives and obstetricians had 5-11 and 10-30 years of experience, respectively. All obstetricians had expertise in maternal-fetal medicine and participate in both undergraduate and resident education. All participants worked clinically at the same department, as it was the only Swedish department undertaking obstetric triage at the time of the study. The participants received no financial compensation.

### The Delphi process

A four-round modified Delphi process was conducted during 2021-2023. Table 2 presents a summary of the process. Supplementary table 1 constitutes a more detailed presentation. All participants were informed about the study purpose and the voluntary nature of participation. Absence from one round excluded further participation. Answering the questionnaire anonymously (round 1) was considered equivalent to consenting to participate. For the following Delphi rounds, written informed consent was given. Due to the aim of the discussions and the different professions’ responsibilities in the ED, the midwives did not participate beyond round 1.

**Table 2:** Overview of the four-round modified Delphi process

**Participants**	**Format**	**Action/Question**	**Result**	**Degree of consensus**
***Round 1***				
Seven senior obstetricians Three senior midwifes	Survey by e-mail	Which condition/s are you concerned about when a patient presents with these symptoms?	63 conditions identified	.
LL	Compilation	.	Grouping conditions, see Supplementary 1	.
***Round 2***				
Six senior obstetricians	Discussion in physical meeting	“*If your assessment of the patient at the ED reveals a severe and/or urgent case of any of the 63 conditions, which intervention/treatment would you initiate at the ED that you* would not initiate* if the case was less severe/urgent”?*	40 interventions identified	100%
LL + VS	Discussion	Compilation of outcome measurements excluding outcome measurements defined by/initiated in accordance with GOTS.	30 interventions / outcome measurements	.
***Round 3***				
Six senior obstetricians	Discussion by e-mail	Discussion on suggested changes, such as removed intervention	31 interventions / outcome measurements	100%
LL + VS + EE	Discussion	Grouping and further assessment of outcome measurements		.
***Round 4***				
Four senior obstetricians	Anonymous grading Discussion in physical meeting	Grading of interventions / outcome measurements	3 levels of interventions, see Table 3	100%

LL, VS, EE, - initials of the authors, *ED* emergency department, *GOTS* Gothenburg obstetric triage system

Participants where obstetricians and midwives working at the obstetric ED at the Obstetric unit of Sahlgrenska University Hospital where triage is based on GOTS. For detailed process description, see Supplementary 1.

#### Round 1

A written invitation to participate was sent by e-mail to eligible participants. All obstetricians (7/7) and three midwives (3/7) chose to participate. After initial consent was provided, an online questionnaire was distributed. The questionnaire was based on patient charts from 380 consecutive visits to the ED, identifying the most common signs and symptoms of the patients attending the obstetric ED. It contained descriptions of the most urgent presentations of these symptoms, such as “gestational week < 34 and ongoing vaginal bleeding and/or contractions” or “decreased fetal movements and no detectable heart rate”. For a full description of signs and symptoms, see Supplement 2. Each description was assessed with the question, *“Which conditions or diagnoses are you concerned about in a patient with these symptoms?”*. Participants answered with unlimited free text. Sixty-three conditions or diagnoses were identified in round 1; they were subsequently summarised in a document for round 2 (see Supplement 3).

#### Round 2

A new invitation for a physical meeting was sent to the obstetricians participating in round 1. The question to be addressed was: “*If your assessment of the ED patient reveals a severe and/or urgent case of any of the 63 conditions, which intervention/treatment would you initiate at the ED that you would not initiate if the case were less severe/urgent?”* Midwives were excluded from further participation as initiating interventions/treatments for patients presenting with severe and/or urgent symptoms is not within their profession’s task or competence.

All but one obstetrician attended the meeting. The discussion was held in a comfortable environment and was moderated by the first author (LL), to ensure that all participants took part in the discussion. The discussion proceeded until the predetermined level of 100% consensus was achieved, meaning that all participants were in total agreement regarding relevant intervention/treatment*.* Forty interventions were identified.

Two of the authors (LL and VS) reviewed all interventions in order to remove interventions already included in the GOTS, such as basic laboratory testing or cardiotocogram (CTG) in specific conditions.

#### Round 3

The 30 remaining interventions and the rationales for removing the others (Supplement 1) were sent by email to the six obstetricians participating in round 2 for assessment and confirmation. Again, 100% consensus was achieved.

#### Round 4

While all obstetricians agreed that the 30 interventions were potentially life-saving, it was evident that some interventions were directly life-saving while other interventions were needed to differentiate life-threatening conditions from less severe conditions. Thus, interventions corresponding to directly life-saving procedures needed to be classified differently from interventions that ruled out more severe conditions. Therefore, a fourth and final round was initiated. With the 30 interventions serving as outcome measures for urgency in triage, the final round aimed to further specify the interventions’ significance and correlate them to different levels of acuity. A new invitation was sent to the six participating obstetricians from round 3, and four were able to participate. The interventions were anonymously graded at one of three levels: 1) not directly lifesaving/preventing lasting morbidity, 2) potentially lifesaving/preventing lasting morbidity and 3) directly lifesaving/preventing lasting morbidity. This was followed by discussion until 100% consensus was reached. This grading led to the division of one previous intervention into two due to two different acuity levels, finally resulting in 31 interventions.

## Results

Thirty-one interventions were defined (Table 3) and weighted in three levels representing different levels of clinical urgency. Interventions range from e.g. cardiopulmonary resuscitation and perimortem caesarean sectio (reflecting the most severe conditions classified as directly lifesaving/preventing lasting morbidity), antibiotic infusion or atisoban intravenously (reflecting interventions that are potentially lifesaving/preventing lasting morbidity) to repeated laboratory investigations within six hours or surveillance of blood pressure and altered hypertensive medication orally (reflecting interventions that are not directly lifesaving/preventing lasting morbidity). In addition, the interventions are differentiated to either be performed regardless of admittance to the hospital or in relation to admittance to the hospital. This differentiation is performed to avoid influence of contextual, organizational factors. It also differentiates patients in need of admittance for normal conditions such as need of pain relief in active stage of labor from patients with an urgent medical condition.

**Table 3:** Grading of outcome measures

**Type of intervention**	**Grading by severity**		
	**Directly lifesaving / preventing serious adverse event 3**	**Potentially lifesaving / potentially preventing serious adverse event 2**	**Not directly lifesaving / preventing serious adverse event 1**
**Actions and examinations *performed at the ED or ordered from ED and performed in direct relation to admission***			
*Overall*	Cardiopulmonary rescue (CPR)		
	Diagnosing Intrauterine fetal death (IUFD)		
*Surgery*	Perimortem cesarean sectio	Operation within 30 min	
	Immediate cesarean sectio		
*Further examinations*		Radiology – computer tomography (CT) / magnetic resonance imaging (MRI) / ultrasound	Echocardiogram (ECG)***
		Thromboelastography (TEG)	Repeated laboratory investigations within 6 hours
		Blood gas	
		Cardiac enzymes	
**Admission to hospital *and* one or more of the *immediate* interventions listed below**			
*Failing vital functions*	ICU*	CICU**	
	Continuous positive airway pressure (CPAP)	Inhalation and/or oxygen	
	Naloxone - suspected morphine overdose	Diuretics iv	
*Venous thromboembolism*	Thrombolysis	Low molecular weight heparin (LMWH) - initiating treatment of suspected or verified venous thrombosis	
*Eclampsia/preeclampsia/ hypertension*	Hypertensive medication infusion		Surveillance of blood pressure and altered hypertensive medication orally
	Magnesium infusion - treatment of eclampsia	Magnesium infusion - prophylactic in severe preeclampsia	
*Infection*		Antibiotic infusion	
*Allergic reaction*	Adrenaline intravenously (iv) / intramuscular (im) - treatment of severe allergic reaction		Cortisone orally
*Symptomatic relief*			Analgesic by morphine and/or hyoscinbutylbromid and/or non-steroidal anti-inflammatory drugs (NSAID)
			Blood transfusion
*Fetal*	Magnesium infusion - neuroprotective in prematurity	Continuous cardiotocogram (CTG)	
		Atisoban intravenous	
		Cortisone intramuscular	

^*^ICU – intensive care unit – admission when failing vital functions, possibly imminent need of intubation

^**^CICU – cardiac intensive care unit – admission when suspected or established heart failure. Enables immediate ECG

^***^Ordered from the ED and performed subacute within 24 hours

*ED* Emergency department

## Discussion

To the best of our knowledge, this is the first study to establish surrogate outcome measures for obstetric triage enabling construct validation of an OTS. Until now, there has been no unanimous concept to scientifically validate or compare OTSs. The study presents a modified Delphi process, resulting in a set of 31 outcome measures graded at three acuity levels.

When discussing the findings, it is important to keep in mind that the purpose of triage is to identify and prioritise patients with urgent conditions in order to provide timely interventions, not to predict patient outcome. Indeed, validating triage systems is challenging as there is no definition of true acuity*.* When lacking a “gold standard”, construct validity is applied for validation, facilitated by surrogate outcome measures. However, the appropriateness of outcome measures such as mortality in the ED, admission to hospital, LOS in the ED, resource utilisation and costs is debateable, especially as context and available resources are rarely specified. For example, resource utilisation may be greatly affected by local circumstances such as access to ultrasound competence or timely laboratory investigations. Moreover, several external factors may affect outcomes after patients have passed through triage, e.g. number of available beds. Thus, generalisability from studies using such outcome measures may be limited.

A previous study on the Obstetrical Triage Acuity Scale (OTAS) showed failure of the system to predict admission to hospital [21]. Similarly, three systematic reviews on non-OTSs found insufficient evidence that mortality or admission to hospital were outcome measures that reflected acuity, particularly at the lower triage levels, as well as identifying several negative issues in EDs when triage results in incorrect prioritization of patients [13, 28, 29]. While admission to ICU and admission to hospital are included as outcome measures in this study as well, the key difference is combining admission with an intervention. Many obstetric patients may present with a high need for admission without being ill – a patient going into labour (>33+6 weeks) needs transfer to a labour unit to access qualified care, including pain relief. Nevertheless, this patient should be triaged to a lower level of acuity than a patient with severe preeclampsia in need of antihypertensive medication. Both patients will be admitted, but only the latter will receive an intervention as defined in this study.

Commonly used measures, such as resource utilisation and LOS in the ED imply a predictive ability of the triage system. However, predicting outcomes is not a triage system’s primary aim. If the treatment/intervention following an accurate triage process is successful, the patient may not require admission and mortality may be avoided. Therefore, outcome measures must, as far as possible, reflect the degree of urgency in immediate or closest proximity to the time of triage. With clinically relevant outcome measures differentiated into three levels of urgency, a validation of an OTS will presumably have a higher likelihood of evaluating the OTS’s ability to discriminate patients into different levels of acuity. Only when assured that a triage system is valid can patient safety be warranted. Likewise, only then may outcome measures related to planning resource utilisation and costs at the organisational level be of interest. To apply a triage system predictively, its validity must be addressed with the question “Are we identifying the right patients?” If not, organisational planning is likely to be based on irrelevant information.

Ideally, a set of outcome measures would be applicable for every OTS and clinical setting. This study presents a modified Delphi process identifying a set of outcome measures for obstetric triage, influenced to a limited extent by external factors. The outcome measures were adapted to the currently implemented OTS as it specifies initial treatment and interventions such as laboratory investigations. In addition, the outcome measures do not include vital sign parameters as these are an integrated part of the OTS itself. Vital sign parameters are fundamental in triaging obstetric patients and should be used as a part of the triage process itself, not as an outcome measure. Nevertheless, by visualising the process and the outcome measures themselves, the latter may be used as a reference standard or template for comparative studies of OTSs implemented in similar contexts.

Every ED has its local conditions and different OTSs may include or exclude initial management steps, careful assessment of whether the outcome measures are applicable in each setting is necessary. Otherwise, we propose that a similar process of establishing acuity outcome measures should be undertaken when an OTS is implemented in a new setting, enabling meaningful validation.

### Strengths and limitations

This paper describes the transparent process that generated outcome measures specific enough to serve as a foundation for comparative studies on obstetric triage in similar contexts.

To the best of our knowledge, this is the first study to establish surrogate outcome measures for obstetric triage and thereby enabling construct validation and comparisons of OTS. The modified Delphi process was based on actual patients seeking emergency care at the unit and all the participants have extensive clinical experience and knowledge on obstetric patients seeking acute care, granting high face validity. Consensus building techniques, such as a Delphi process, are applicable when traditionally used, research-based techniques are inapplicable and high face validity is a prerequisite for potential further development of the outcome measures.

The process was context-related, which may have reduced generalisability. Nevertheless, in contexts like the Swedish healthcare system and with similar population and disease-spectrum, the outcome measures should be applicable.

The number of participants in the Delphi process was somewhat small, partly because no other unit in Sweden had implemented obstetric triage as a working method at the time of this study. Inviting additional obstetricians from the same unit was considered superfluous as the included participants represent a diversity in experience and clinical profile. All participants being from the same unit may constitute a bias; the discussion being potentially influenced by a patient management culture and hierarchy. Including clinicians from other clinics might have led to a more diverse discussion. Nevertheless, the Delphi process continued until 100% consensus was reached in rounds 2-4.

## Conclusions

All triage systems should be validated to guarantee patient safety and correctly allocate resources. A structured consensus-building technique such as a modified Delphi process can be used to develop suitable outcome measures with high face validity. The outcome measures created in this study may serve as a template for validating OTSs in similar contexts. Future studies should focus on validating available OTS and testing the developed outcome measures in different clinical setting.

## Supplementary Information

Supplementary Material 1.

Supplementary Material 2.

Supplementary Material 3.

## Data Availability

All data generated or analyzed during this study are included in this published article and its supplementary information files.

## Abbreviations

CTG: Cardiotocogram
ED: Emergency department
ECG: Electrocardiogram
GOTS: Gothenburg Obstetric Triage System
ICU: Intensive care unit
LOS: Length of stay
OTAS: Obstetrical triage acuity scale
OTS: Obstetric triage system
