# Supplementary material for: Validating obstetric triage systems, what are we really measuring - A modified Delphi process introducing outcome measures for obstetric emergency triage systems
Source: BMC Pregnancy Childbirth. 2025 Apr 2;25:383. doi: 10.1186/s12884-025-07476-5 (PMC11963699; doi:10.1186/s12884-025-07476-5)
Supplement: Supplementary file 2 — Supplementary Material 2. [file 12884_2025_7476_MOESM2_ESM.docx]

**Supplement 2** The most common signs and symptoms when attending the obstetric ED including the highest degree of acuity of these conditions, consecutive obstetric ED visits during two weeks (n =380)

**Contractions**

Imminent/threatened delivery < gestational week (gw) 34; fetal parts in vagina < gw 34+0 with regular contractions less than five minutes apart, with or without bleeding

**Rupture of membranes**

< gw 34+0, with bleeding and/or contractions

**Vaginal bleeding**

Ongoing profuse vaginal bleeding, with or without increased uterine tone

**Decreased fetal movements or abnormal fetal heart trace**

No fetal movements and preterminal or pathological cardiotocogram (CTG), umbilical cord prolapse, no detectable fetal heart trace, no fetal movements and abnormal CTG

**Hypertension or suspected/known preeclampsia – found at other health institution**

Eclampsia/convulsions; hypertension or preeclampsia in combination with confusion; systolic blood pressure > 160 mmHg and/or diastolic blood pressure > 110 mmHg, with or without preeclampsia symptoms; systolic blood pressure > 180 mmHg and/or diastolic blood pressure > 120 mmHg, with or without preeclampsia symptoms; vomiting in combination with elevated blood pressure

**Headache/neurological symptoms, including altered level of consciousness**

Eclampsia/convulsions, severe sudden headache, altered level of consciousness, neurological deficit, neck rigidity

**Pain in abdomen/back/flank**

Sudden severe and ongoing pain, continuously increased uterine tone

**Postpartum bleeding**

Profuse ongoing bleeding postpartum

**Signs/symptoms of infection**

Signs/symptoms of sepsis e.g., petechiae, fever > 39.0℃ and ongoing or recent chills; immunodeficiency

**Chest pain/ respiratory distress**

Severe chest pain and cold sweat, nausea and/or dyspnea, ongoing or recent altered level of consciousness

**Signs/symptoms of venous thromboembolism**

Swelling of extremity, ongoing dyspnea, ongoing or recent altered level of consciousness
