# Supplementary material for: Validating obstetric triage systems, what are we really measuring - A modified Delphi process introducing outcome measures for obstetric emergency triage systems
Source: BMC Pregnancy Childbirth. 2025 Apr 2;25:383. doi: 10.1186/s12884-025-07476-5 (PMC11963699; doi:10.1186/s12884-025-07476-5)
Supplement: Supplementary file 3 — Supplementary Material 3. [file 12884_2025_7476_MOESM3_ESM.docx]

**Supplement 3** Medical conditions derived from the questionnaire in round 1 of the modified Delphi process

**Cardiac or arterial complications/conditions**

Peripartum cardiomyopathy, myocardial infarction, arrythmia, cardiac tamponade, cardiac arrest, vertebral artery dissection, aortic dissection

**Delivery complications/conditions**

Contractions, uterine rupture, delivery outside labour ward, dystocia, breech /foot-breech presentation

**Fetal/neonatal complications/conditions**

Intrauterine fetal death, intrauterine growth restriction, fetal hypoxia/asphyxia, preterm labour, umbilical cord prolapse, neonatal cerebral palsy

**Haemorrhagic complications/conditions**

Placental abruption – total or partial, placenta previa, vasa previa /fetal haemorrhage, coagulopathy, anaemia, haemorrhagic shock, retained placental residue, arteriovenous malformation, profuse menstruation during puerperium

**Hypertensive disorder**

Severe preeclampsia, HELLP syndrome, eclampsia

**Infectious complications**

Chorioamnionitis, endometritis, Group A streptococcal infection, sepsis, mastitis, meningitis, Covid-19

**Intraabdominal complications/conditions**

Pyelonephritis, hydronephrosis, kidney stones, gallstones, appendicitis, bowel complication such as ileus, intraabdominal haemorrhage, peritonitis

**Intracranial complications/conditions**

Stroke, epilepsy, migraine, other intracranial condition

**Other complications/conditions**

Organ failure, ketoacidosis, intoxication, Korsakoff-Wernicke syndrome, pulmonary oedema, leukaemia, severe asthma, severe allergy, maternal death

**Thromboembolic event**

Pulmonary embolism, portal vein thrombosis, pelvic vein thrombosis, other deep vein thrombosis
